# CYLD Deubiquitinase Negatively Regulates High Glucose-Induced NF-*κ*B Inflammatory Signaling in Mesangial Cells

**DOI:** 10.1155/2017/3982906

**Published:** 2017-11-12

**Authors:** Yanhui Li, Wei Huang, Youhua Xu, Luping Zhou, Yaling Liang, Chenlin Gao, Yang Long, Yong Xu

**Affiliations:** ^1^Department of Endocrinology, The Affiliated Hospital of Southwest Medical University, Luzhou, Sichuan 646000, China; ^2^Department of Kidney Endocrinology, The People's Hospital of Qingbaijiang, Qingbaijiang, Sichuan 610300, China; ^3^Faculty of Chinese Medicine, Macau University of Science and Technology, Avenida Wai Long, Taipa, Macau; ^4^State Key Laboratory of Quality Research in Chinese Medicine, Macau University of Science and Technology, Avenida Wai Long, Taipa, Macau; ^5^Collaborative Innovation Center for Prevention and Treatment of Cardiovascular Disease of Sichuan Province, Southwest Medical University, Luzhou, Sichuan 646000, China

## Abstract

Nuclear factor-kappa B (NF-*κ*B) is the key part of multiple signal transduction of inflammation in the pathogenesis of diabetic nephropathy (DN). The ubiquitin-proteasome system is extensively involved in the regulation of the NF-*κ*B pathway. Cylindromatosis (CYLD) has deubiquitinase activity and acts as a negative regulator of the NF-*κ*B signaling pathway. However, the association between CYLD and NF-*κ*B inflammatory signaling in DN is unclear. In the present study, mouse glomerular mesangial cells (GMCs) and rat GMCs were stimulated by elevated concentrations of glucose (10, 20, and 30 mmol/L high glucose) or mannitol as the osmotic pressure control. CYLD was overexpressed or suppressed by transfection with a CYLD expressing vector or CYLD-specific siRNA, respectively. Our data showed that high glucose significantly inhibited the protein and mRNA expression of CYLD in a dose- and time-dependent manner (both *p* < 0.05). siRNA-mediated knockdown CYLD facilitated the high glucose-induced activation of NF-*κ*B signaling and triggered the release of MCP-1, IL-6, and IL-8 (all *p* < 0.05). However, these high glucose-mediated effects were blunted by overexpression of CYLD (*p* < 0.05). The present results support the involvement of CYLD in the regulation of NF-*κ*B inflammatory signaling induced by elevated glucose, implicating CYLD as a potential therapeutic target of DN.

## 1. Introduction

Diabetic nephropathy (DN) is a common and serious diabetic microvascular complication. Nuclear factor *κ*B (NF-*κ*B) plays a central regulatory role in the expression of various inflammatory cytokines and adhesion molecules involved in the occurrence of DN [1]. In unstimulated cells, NF-*κ*B is conjugated to I*κ*B*α* and kept in an inactive state in the cytosol [2]. Different physiological or pathological stimuli can activate and promote the phosphorylation of I*κ*B*α* and its subsequent degradation by the ubiquitin-proteasome pathway (UPP), thereby exposing the nuclear-localization sequence (NLS) of NF-*κ*B and leading to its translocation from the cytoplasm to the nucleus, where it activates the transcription of genes for the immune and inflammatory response [3].

Ubiquitin targets proteins for degradation by the 26S proteasome. Our previous research proved that ubiquitylation plays an important role in the activation of the NF-*κ*B signaling pathway in the pathogenesis of DN [4]. Like phosphorylation, ubiquitination is a reversible reaction mediated by deubiquitinases (DUBs), which hydrolyze ubiquitin chains and which are considered to oppose the functions of their counteractive ubiquitinases [5]. More than 90 DUBs exist in the human genome, and some DUBs are specific for distinct ubiquitin linkages, suggesting that DUBs participate in specific biological functions.

As one of the DUBs, cylindromatosis (CYLD) is the main negative regulatory factor and inflammation inhibiting factor in the NF-*κ*B signaling pathway. CYLD is activated by different inducers, including tumor necrosis factor-alpha (TNF-*α*), interleukin-1 (IL-1), cluster of differentiation 40 (CD40), and phorbol 12-myristate 13-acetate (PMA) [6]. However, subsequent studies have indicated that although CYLD targets NF-*κ*B signaling factors, its function may depend on the cell type and stimulating receptor [7]. Furthermore, there is no evidence to support the association between CYLD and NF-*κ*B signaling in the pathogenesis of DN.

In the present study, we observed the expressions of CYLD, I*κ*B*α*, phosphorylated (p)-I*κ*B*α*, NF-*κ*Bp65, and p-NF-*κ*Bp65 and the release of MCP-1, IL-6, and IL-8 in cultured glomerular mesangial cells (GMCs) stimulated by elevated concentrations of glucose, siRNA, or lentivirus vector constructed to realize silencing or overexpression of CYLD gene. These experiments were done to explore the role of CYLD in the regulation of NF-*κ*B inflammatory signaling in the pathogenesis of DN.

## 2. Materials and Methods

### 2.1. Cell Culture and Treatment

Mouse GMCs (SV40 MES 13) and rat GMCs (HBZY-1) were purchased from the China Center for Type Culture Collection (CCTCC) and maintained in low glucose (5.6 mmol/L) Dulbecco's Modified Eagle Medium (DMEM) supplemented with 10% fetal bovine serum (Hyclone) at 37°C and 5% CO_2_. The experimental groups included normal control group (NC; medium with 5.6 mmol/L glucose), high glucose treatment group (HG; culture medium with 10 mmol/L (HG1), 20 mmol/L (HG2), or 30 mmol/L (HG3) glucose), osmotic pressure control group (OP; medium with 5.6 mmol/L glucose + 24.4 mmol/L mannitol), and MG132 intervention group (with medium that contained 30 mmol/L glucose + 1 *μ*mol/LMG132, in which MG132 was added to the culture medium to block protein ubiquitination). Cells were exposed to these treatments for 6, 12, 24, 48, and 72 h; protein and mRNA were extracted and culture supernatants were collected for further study.

### 2.2. CYLD Overexpression Vector Construction and Transfection

Mouse CYLD gene primers were designed and the* cyld* gene was obtained by polymerase chain reaction (PCR) amplification. A recombinant lentivirus vector harboring* cyld* was generated by restriction enzyme (Jikai, Shanghai, China) action on the specific domain and transformation between* cyld* and vector. Lentivirus was purified and packaged using a packaging mixture (Jikai). The HEK293 human embryonic kidney T cells were transferred with the packaged lentivirus and the multiplicity of infection (MOI) was detected by fluorescence microscopy to optimize the infection conditions. Stable overexpression of CYLD (LV-CYLD group) was achieved using medium containing 1 × 10^8^ transducing units (TU)/mL of CYLD lentivirus (GMC MOI = 50). The blank transfection group (LV-CON235 group) used medium with 1 × 10^8^ TU/ml CON235 virus (GMC MOI = 50). Both were stimulated using 30 mmol/L glucose for 24 h at 37°C. Cells and culture supernatants were collected for Western blotting and ELISA analyses.

### 2.3. siRNA Transfection

siRNA targeting CYLD (sense: 5′-GAG GAT CCC CGG GTA CCG GTC GCC ACC ATG AGT TCA GGC CTG TGG AGC CAA G-3′; anti-sense: 5′-TCC TTG TAG TCC ATA CCT TTG TAC AGG CTC ATG GTT GGA CTC-3′) were synthesized by RiboBio Biotechnology (Guangzhou, China). Transfection was done using Lipofectamine® 2000 (Invitrogen, Karlsruhe, Germany) following the manufacturer's instructions. Experiments were performed at 48 h after transfection. siRNA-mediated knockdown of CYLD was achieved by growth of cells in medium containing 100 nmol/L CYLD siRNA. These cells were then stimulated by 30 mmol/L glucose. Cells and culture supernatants were collected for Western blotting and ELISA analyses.

### 2.4. Protein Extraction and Western Blot

Total proteins were isolated from GMCs using a total protein extraction kit (Beyotime, Beijing, China). Proteins were separated by sodium dodecyl sulfate-polyacrylamide gel electrophoresis and transferred to a polyvinylidene difluoride (PVDF) membrane (Millipore, Billerica, MA). Immunoblotting was performed using anti-CYLD goat polyclonal antibody (1 : 1,000; Abcam, Cambridge, MA), anti-I*κ*B*α* mouse monoclonal antibody (1 : 1,000; CST, Boston, USA), anti-p-I*κ*B*α* ser32/36 mouse monoclonal antibody (1 : 1,000; CST, Boston, USA), anti-NF-*κ*Bp65 mouse monoclonal antibody (1 : 2,000; CST, Boston, USA), anti-p-NF-*κ*Bp65 ser536 mouse monoclonal antibody (1 : 2,000; CST, Boston, USA), and anti-*β*-actin rabbit monoclonal antibody (1 : 3000; Beyotime, Beijing, China). Images were taken with a molecular imaging system (FUJI, Tokyo, Japan).

### 2.5. RNA Extraction and Reverse-Transcription- (RT-) PCR

Total RNA was extracted from GMCs using an RNA extraction kit (Tiangen Biotech, Beijing, China). Total RNA was reverse-transcribed using an RNA PCR kit (Baoshengwu, Dalian, China). cDNA was amplified in a gradient thermal cycler (Eppendorf, Hamburg, Germany) using PCR Master Mix (Baoshengwu). The results were determined using an ultraviolet transilluminator and normalized to glyceraldehyde 3-phosphate dehydrogenase (GAPDH) gene expression. The primer sequences were CYLD (forward, 5′-CTT GCC TGA CTG GGA CT-3′; reverse, 5′-TTC TGA CCA CCA TCT CG-3′) and GAPDH (forward, 5′-TGG CCT TCC GTG TTC CTA C-3′; reverse, 5′-GAG TTG CTG TTG AAG TCG CA-3′).

### 2.6. Enzyme-Linked Immunosorbent Assay (ELISA)

GMCs (1 × 10^4^ per well) were seeded in 24-well plates and induced by high glucose as described above. MCP-1, IL-6, and IL-8 protein level in the culture supernatants were determined using commercially available ELISA kits (Jikai, Shanghai, China) according to the manufacturer's protocols. MCP-1, IL-6, and IL-8 protein levels were determined by comparing the samples to the standard curve generated by the kit.

### 2.7. Statistical Analysis

All data are obtained from at least three independent experiments and are expressed as mean ± standard deviation (SD). Differences were statistically analyzed using one-way analysis of variance (ANOVA), followed by the Least Significant Difference post hoc test for multiple comparisons. A probability value of *p* < 0.05 was considered significant.

## 3. Results

### 3.1. High Glucose Inhibits the Expression of CYLD in GMCs

**Figure 1:**
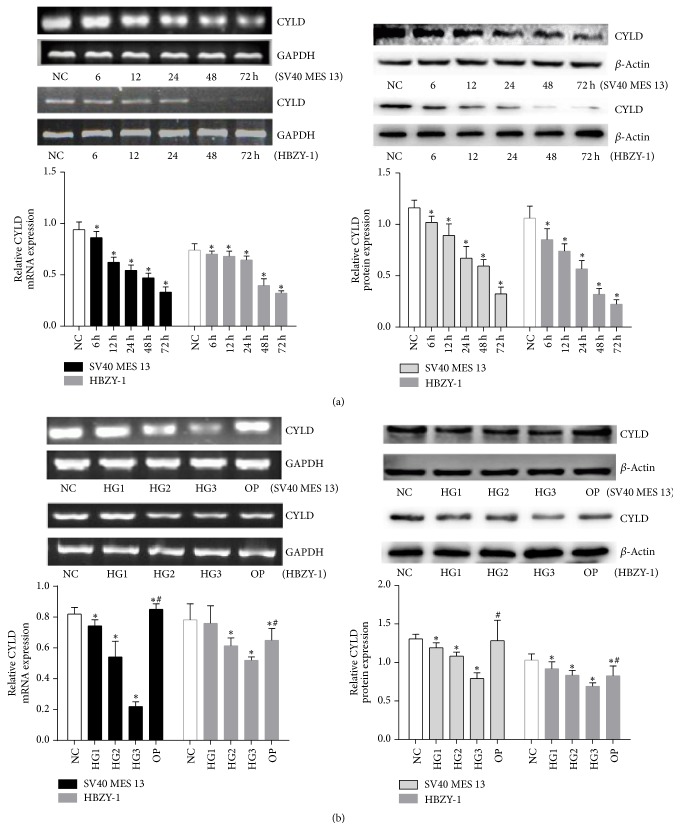
*CYLD protein and mRNA expression after high glucose challenge for various times and various glucose concentrations determined by Western blot and RT-PCR*. (a) GMCs (SV40 MES 13 and HBZY-1) were treated with 30 mmol/L glucose for 6, 12, 24, 48, and 24 h, and Western blot and RT-PCR were performed to detect the expression of CYLD. (b) GMCs (SV40 MES 13 and HBZY-1) were treated with the indicated concentrations of glucose or mannitol for 24 h. The gray graph shows the relative statistical values for CYLD protein and mRNA expression in each group. The data were normalized to GAPDH/*β*-actin and are expressed as mean ± SD; ^*∗*^*p* < 0.05 compared with NC group; ^#^*p* < 0.05 compared with 30 mmol/L glucose (HG3) group.

To determine whether CYLD is regulated by glucose in GMCs, we first detected CYLD proteins and mRNA by Western blot and RT-PCR. As shown in Figure 1(a), compared with the NC group, the relative CYLD mRNA and protein expressions in GMCs (SV40 MES 13 and HBZY-1) gradually decreased after treatment with 30 mmol/L glucose for 6 h to 72 h and were lowest at 72 h (*p* < 0.05). Compared with the NC group, the relative CYLD mRNA and protein expressions were inhibited by different concentrations of high glucose at 24 h, particularly in the 30 mmol/L glucose group (*p* < 0.05; Figure 1(b)). However, there was no significant change between the NC and OP groups, suggesting that osmotic pressure had little effect on the inhibited expression of CYLD in the high glucose medium. These data suggest that high glucose inhibited CYLD expression in GMCs (SV40 MES 13 and HBZY-1) in a time- and dose-dependent manner.

### 3.2. High Glucose Induced the Activation of NF-*κ*B Inflammatory Signaling by Phosphorylation and Ubiquitination Degradation of I*κ*B*α*

**Figure 2:**
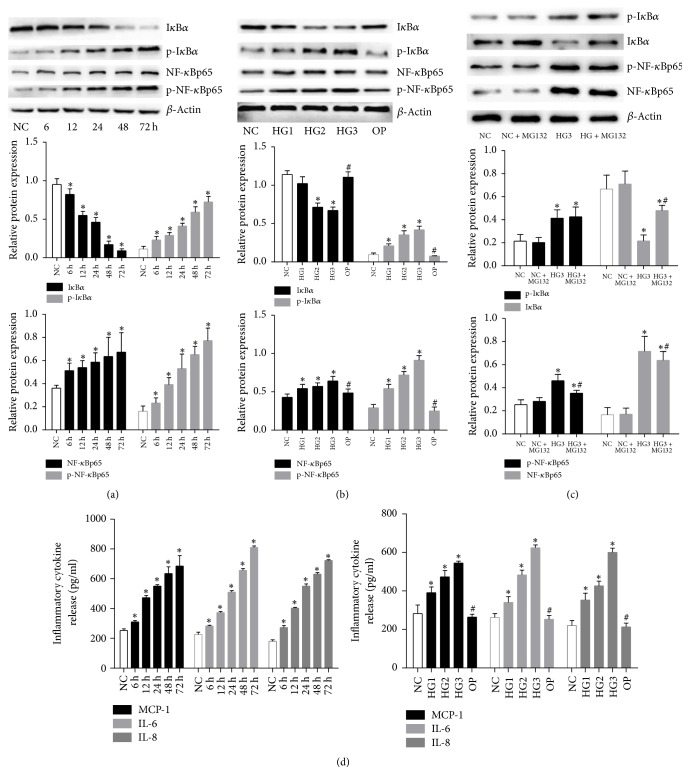
*High glucose induces the activation of NF-κB signaling and release of MCP-1, IL-6, and IL-8*. (a) Protein expressions of I*κ*B*α*, p-I*κ*B*α*, NF-*κ*Bp65, and p-NF-*κ*Bp65 in lysates of mouse GMCs treated with 30 mmol/L glucose for 6, 12, 24, 48, and 72 h were detected by Western blot. (b) Protein expressions of I*κ*B*α*, p-I*κ*B*α*, NF-*κ*Bp65, and p-NF-*κ*Bp65 in lysates of mouse GMCs treated with an indicated concentration of high glucose for 24 h as detected by Western blot. (c) MG132 partially reversed high glucose-induced I*κ*B*α* ubiquitination degradation and NF-*κ*B activation. (d) Release of the inflammatory cytokines MCP-1, IL-6, and IL-8 in the cell culture supernatant was quantified by ELISA. Data were normalized with respect to *β*-actin and are expressed as mean ± SD. The gray graphs show the relative statistical values in each group and confirmed these trends. ^*∗*^*p* < 0.05 compared with NC group; ^#^*p* < 0.05 compared with 30 mmol/L glucose (HG3) group.

Compared with the NC group, the protein expressions of p-I*κ*B*α*, NF-*κ*Bp65, and p-NF-*κ*Bp65 were significantly induced following 6, 12, 24, 48, and 72 h of exposure to 30 mmol/L glucose in a time-dependent manner in mouse GMCs (SV40 MES 13) (*p* < 0.05; Figure 2(a)). The protein levels of these signaling molecules were also significantly induced by the different concentrations of high glucose in a dose-dependent manner (*p* < 0.05; Figure 2(b)). However, as an important inhibitory protein of NF-*κ*B pathway, the levels of I*κ*B*α* protein were significantly decreased by high glucose in time- and dose-dependent manners (*p* < 0.05; Figures 2(a) and 2(b)). After the proteasome inhibitor MG132 treatment, I*κ*B*α*, p-NF-*κ*Bp65 and NF-*κ*Bp65 protein levels were partially reversed compared with the HG3 group (*p* < 0.05), but p-I*κ*B*α* protein levels were not changed significantly (*p* > 0.05). Moreover, no apparent differences were found between the NC group and NC + MG132 group, suggesting that MG132 partially reversed high glucose-induced I*κ*B*α* ubiquitination degradation and NF-*κ*B activation (Figure 2(c)). Next, we used ELISA to determine whether high glucose could stimulate the release of MCP-1, IL-6, and IL-8, which are downstream inflammatory cytokines of NF-*κ*B signaling pathway. As shown in Figure 2(d), compared with the NC group, levels of MCP-1, IL-6, and IL-8 protein in culture supernatants were significantly increased by high glucose in time- and dose-dependent manners (*p* < 0.05). The collective data indicate that high glucose induced the activation of NF-*κ*B inflammatory signaling by phosphorylation and ubiquitination degradation of I*κ*B*α*.

### 3.3. siRNA-Mediated Knockdown CYLD Facilitates the Activation of NF-*κ*B Induced by High Glucose

**Figure 3:**
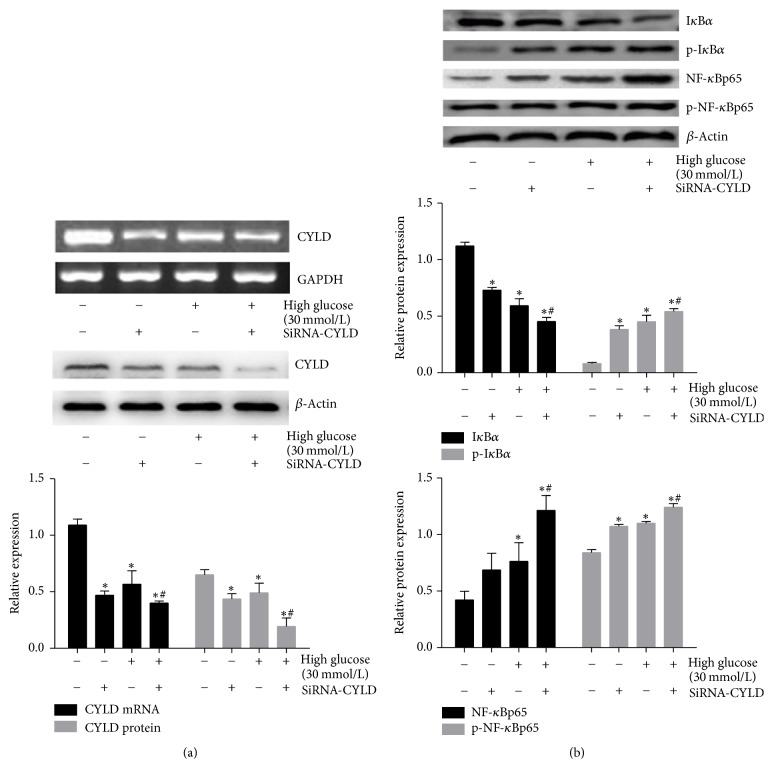
*siRNA-mediated knockdown CYLD facilitated the activation of NF-κB induced by high glucose*. (a) RT-PCR and Western blot detected the mRNA and protein expression of CYLD in mouse GMCs treated with 30 mmol/L glucose and (or) CYLD siRNA for 24 h. (b) Western blot detection of the expression of I*κ*B*α*, p-I*κ*B*α*, NF-*κ*B, and p-NF-*κ*B in mouse GMCs treated with 30 mmol/L glucose and (or) CYLD siRNA for 24 h. Data were normalized with respect to *β*-actin and are expressed as mean ± SD. The gray graphs show the relative statistical values in each group and confirmed these trends. ^*∗*^*p* < 0.05 compared with the NC group; ^#^*p* < 0.05 compared with the 30 mmol/L glucose stimulation group.

We then investigated the underlying mechanism by which CYLD deubiquitinase was involved in the regulation of NF-*κ*B inflammatory signaling in mouse GMCs. siRNA were constructed to realize silencing CYLD gene; the results showed that compared with NC or the 30 mmol/L glucose group, the protein and mRNA expressions of CYLD were significantly decreased by siRNA-CYLD (*p* < 0.05), suggesting that high-induced downregulation of CYLD was significantly facilitated by transfecting siRNA-CYLD (Figure 3(a)). Meanwhile, Western blot revealed that, compared with the 30 mmol/L glucose group, the level of I*κ*B*α* protein was decreased by siRNA-CYLD (*p* < 0.05), but the protein expressions of p-I*κ*B*α*, p-NF-*κ*Bp65, and NF-*κ*Bp65 were increased (*p* < 0.05) (Figure 3(b)), suggesting that the high glucose-induced the activation of NF-*κ*B was significantly facilitated by synergistically treating siRNA-CYLD.

### 3.4. Overexpression of CYLD Reverses High Glucose-Induced Activation of NF-*κ*B Signaling Pathway

**Figure 4:**
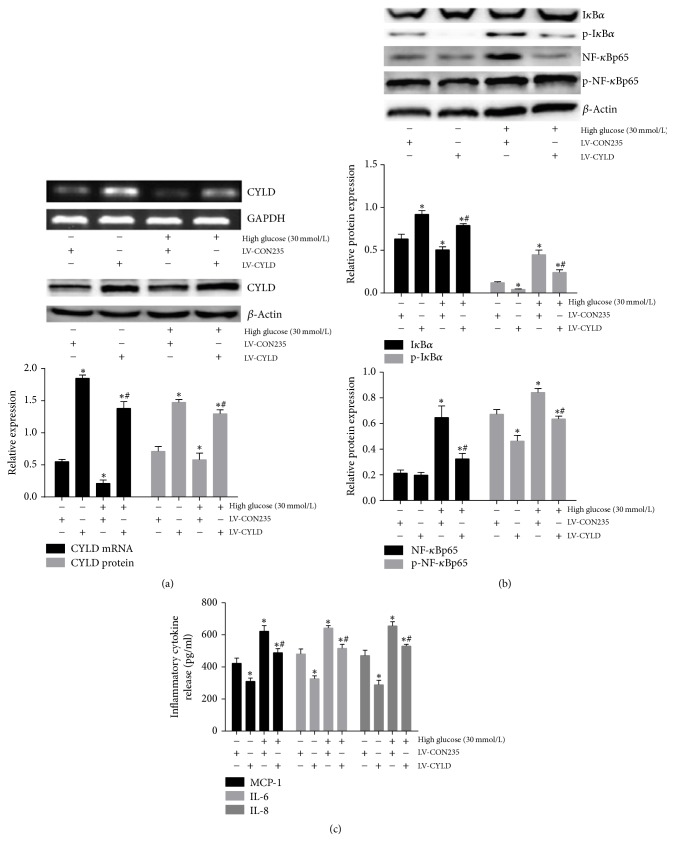
*Overexpression of CYLD reverses high glucose-induced activation of the NF-κB signaling pathway*. (a) RT-PCR and Western blot detection of the mRNA and protein expression of CYLD in mouse GMCs treated with 30 mmol/L glucose and (or) overexpression of CYLD for 24 h. (b) Western blot detection of the expression of I*κ*B*α*, p-I*κ*B*α*, NF-*κ*Bp65, and p-NF-*κ*Bp65 in mouse GMCs treated with 30 mmol/L glucose and (or) overexpression CYLD for 24 h. (c) ELISA detection of the release of the inflammatory cytokines MCP-1, IL-6, and IL-8 from mouse GMCs treated with 30 mmol/L glucose and (or) overexpression of CYLD for 24 h. The gray graphs show the relative statistical values in each group and confirmed these trends. ^*∗*^*p* < 0.05 compared with the NC + blank load transfection (NC + LV-CON235) group; ^#^*p* < 0.05 compared with 30 mmol/L glucose + LV-CON235 group.

To assess the involvement of CYLD deubiquitinase in the regulation of NF-*κ*B signaling, we established the lentivirus vector conferring CYLD overexpression upon transfection of mouse GMCs. Some mouse GMCs were also stimulated by 30 mmol/L glucose for 24 h. As shown in Figure 4(a), the mRNA and protein expression levels of CYLD were significantly increased by CYLD overexpression compared with blank load transfection group (*p* < 0.05). The high glucose-induced decrease of CYLD expression was reversed by overexpression of CYLD (*p* < 0.05), as was high glucose-inhibited I*κ*B*α* protein expression (*p* < 0.05; Figure 4(b)). Conversely, high glucose-induced p-I*κ*B*α*, NF-*κ*Bp65, and p-NF-*κ*Bp65 protein levels were significantly blunted by overexpression of CYLD (Figure 4(b)), and similar results for the downstream inflammatory cytokines MCP-1, IL-6, and IL-8 were observed by ELISA (Figure 4(c)). These results revealed that high glucose-mediated activation of NF-*κ*B inflammatory signaling was blunted by overexpression of CLYD, suggesting that CYLD deubiquitinase negatively regulated NF-*κ*B inflammatory signaling in GMCs.

## 4. Discussion

NF-*κ*B inflammatory signaling has an important role in the occurrence and development of DN [8]. The activation of NF-*κ*B is mediated by the polyubiquitylation of phosphorylated I*κ*B*α*, followed by their proteasomal degradation [4, 9, 10]. We have shown that high glucose can activate NF-*κ*B signaling through the phosphorylation and then ubiquitination of I*κ*B*α*. Moreover, high glucose may be involved in the pathogenesis of DN by specifically impacting I*κ*B*α* sumoylation [11]. The proteasome inhibitor, MG132, ameliorates kidney lesions and attenuates DN by inhibiting I*κ*B*α*, SnoN, and Smad7 protein ubiquitination degradation [12, 13]. These studies suggest that targeting the UPP may be a potential target for the treatment of DN.

Ubiquitination is a dynamic process that can be counterbalanced by deubiquitinating enzymes including the tumor suppressor CYLD [6]. The human CYLD gene is located on chromosome 16q12.1 and encodes a protein of 956 amino acids; the C-terminal region of CYLD contains a catalytic domain with sequence homology to ubiquitin-specific proteases (USP) family members [14]. The initial clue to the signaling function of CYLD came from an RNA interference-based functional screening study, which identified CYLD as a DUB that negatively regulates NF-*κ*B activation. Overexpression of CYLD leads to a decrease in NF-*κ*B activity induced by several receptors including TNFR1, CD40, TLR4, EDAR, and LMP1 [15, 16]. The critical role of CYLD in NF-*κ*B regulation suggests the involvement of this DUB in important biological processes. One prominent function of CYLD is the regulation of immune response and inflammation [17]. CYLD negatively regulates the induction of proinflammatory mediators by* Streptococcus pneumoniae *and* Escherichia coli* [18]. CYLD^−/−^ mice do not develop spontaneous tumors; however, they are highly susceptible to dextran sulfate sodium-induced colitis and azoxymethane-induced tumor development [19, 20]. Thus, CYLD plays a critical role in the suppression of tumor proliferation [21]. In addition, CYLD is suggested to be involved in the induction of cell death [22]. Reduced CYLD expression is also shown to increase the survival of several cell types [23, 24]. However, the association between deubiquitinase CYLD and NF-*κ*B signaling in DN remains unclear. In the present study, we found that high glucose dose- and time-dependently downregulated the protein and mRNA expressions of CYLD in GMCs (SV40 MES 13 and HBZY-1) and increased the expression levels of p-I*κ*B*α*, NF-*κ*Bp65, and p-NF-*κ*Bp65, and furthermore induced the release of MCP-1, IL-6, and IL-8. These results showed that both CYLD and NF-*κ*B inflammatory signaling pathway could be regulated by high glucose, suggesting that CYLD could be involved in the regulation of NF-*κ*B inflammatory signaling under high glucose stress.

Since CYLD can cleave K63 in addition to linear linkages, its functions are not defined by the cleavage of linear chains alone. Previous studies reported that CYLD binds to IKK*γ* and its upstream signaling components including TAK1, TRAF2, TRAF6, and RIP1 [14–16]. In particular, CYLD activity interferes with the NF-*κ*B signaling by catalyzing the specific K63-polyUb chains from IKK*γ*, TRAF2, and TRAF6, without affecting the K48-polyUb chains of I*κ*B*α* [25]. However, recent research revealed that CYLD inhibited NF-*κ*B signaling by deconjugating the polyubiquitylation of phosphorylated I*κ*B*α* proteins and rendered resistance to murine hepatocyte death [26]. Whether CYLD inhibits the activation of NF-*κ*B signaling through the deubiquitination of I*κ*B*α* in high glucose conditions is unclear. In this study, high glucose-inhibited expression of I*κ*B*α* was significantly reversed by the overexpression of CYLD, and p-I*κ*B*α*, NF-*κ*Bp65, and p-NF-*κ*Bp65 proteins levels were significantly decreased. In accordance with NF-*κ*Bp65, similar profiles of proinflammatory cytokines MCP-1, IL-6, and IL-8 released from GMCs were reversed by overexpression of CYLD, suggesting that high glucose-induced activation of NF-*κ*B inflammatory signaling was blunted by overexpression of CYLD. On the contrary, compared with high glucose group, the level of I*κ*B*α* proteins was more obviously decreased by synergistically treating siRNA-CYLD, and p-I*κ*B*α*, NF-*κ*Bp65, and p-NF-*κ*Bp65 were evidently increased, suggesting that high glucose-induced activation of NF-*κ*B was facilitated by siRNA-mediated knockdown of CYLD. We speculate that CYLD deubiquitinase negatively regulates NF-*κ*B inflammatory signaling by deconjugating the polyubiquitylation of phosphorylated I*κ*B*α*, followed by inhibiting activation of NF-*κ*B signaling under high glucose environments.

However, it must be pointed out that overexpression of CYLD did not totally reverse high glucose-induced activation of NF-*κ*B signaling, suggesting that there must be other mechanisms involved in the cross-talk of inflammation induced by high glucose. Even so, our study suggest a potential therapeutic target for the inhibition of the NF-*κ*B inflammatory signaling and treatment of DN. An increasing number of studies have reported that the target drugs of CYLD play an important role in the treatment of various disease. A study reported that, as novel MALT1 inhibitors, *β*-lapachone analogs exhibited potent antiproliferative activity and inhibited the cleavage of CYLD mediated MALT1; this may be a promising therapeutic target for the treatment of aggressive subtype of diffuse large B-cell lymphoma [27]. In another study, interference with CYLD completely restored glucocorticoid resistance in children with acute lymphoblastic leukemia (ALL), suggesting that targeting CYLD may be a pharmacological approach to treatments for patients with refractory ALL [28]. In view of the fact that the regulatory mechanisms of NF-*κ*B signaling are extremely complex, the establishment of an animal model for DN and targeting renal CYLD intervention are necessary further studies, which will focus on the interaction among CYLD and inflammatory signaling to clarify the molecular mechanisms in the pathogenesis of DN and other diabetic complications.

In conclusion, the present study found that high glucose significantly inhibited the expression of CYLD and activated NF-*κ*B inflammatory signaling in a dose- and time-dependent manner. These high glucose-induced effects were facilitated by siRNA-CYLD but blunted by overexpression of CYLD. The present results support the involvement of CYLD deubiquitinase in the regulation of NF-*κ*B inflammatory signaling induced by high glucose, implicating CYLD as a potential therapeutic target of DN.

## References

[1] Dyson H. J., Komives E. A. (2012). Role of disorder in I*κ*B-NF*κ*B interaction. *IUBMB Life*.

[2] Schröfelbauer B., Polley S., Behar M., Ghosh G., Hoffmann A. (2012). NEMO Ensures Signaling Specificity of the Pleiotropic IKK*β* by Directing Its Kinase Activity toward I*κ*B*α*. *Molecular Cell*.

[3] Ren Z., Cui J., Huo Z. (2012). Cordycepin suppresses TNF-*α*-induced NF-*κ*B activation by reducing p65 transcriptional activity, inhibiting I*κ*B*α* phosphorylation, and blocking IKK*γ* ubiquitination. *International Immunopharmacology*.

[4] Gao C., Huang W., Kanasaki K., Xu Y. (2014). The role of ubiquitination and sumoylation in diabetic nephropathy. *BioMed Research International*.

[5] Fraile J. M., Quesada V., Rodríguez D., Freije J. M. P., López-Otín C. (2012). Deubiquitinases in cancer: new functions and therapeutic options. *Oncogene*.

[6] Sun S.-C. (2010). CYLD: A tumor suppressor deubiquitinase regulating NF-B activation and diverse biological processes. *Cell Death & Differentiation*.

[7] Lim J. H., Jono H., Komatsu K. (2012). CYLD negatively regulates transforming growth factor-*β*-signalling via deubiquitinating Akt. *Nature Communications*.

[8] Ma J., Wu H., Zhao CY., Panchapakesan U., Pollock C., Chadban SJ. (2014). Requirement for TLR2 in the development of albuminuria, inflammation and fibrosis in experimental diabetic nephropathy. *International Journal of Clinical and Experimental Pathology*.

[9] Won M., Byun H. S., Park K. A., Hur G. M. (2016). Post-translational control of NF-*κ*B signaling by ubiquitination. *Archives of Pharmacal Research*.

[10] Ikeda F. (2015). Linear ubiquitination signals in adaptive immune responses. *Immunological Reviews*.

[11] Huang W., Xu L., Zhou X. (2013). High glucose induces activation of NF-*κ*B inflammatory signaling through I*κ*B*α* sumoylation in rat mesangial cells. *Biochemical and Biophysical Research Communications*.

[12] Huang W., Yang C., Nan Q. (2014). The proteasome inhibitor, MG132, attenuates diabetic nephropathy by inhibiting SnoN degradation in vivo and in vitro. *BioMed Research International*.

[13] Gao C., Aqie K., Zhu J. (2014). MG132 ameliorates kidney lesions by inhibiting the degradation of smad7 in streptozotocin-induced diabetic nephropathy. *Journal of Diabetes Research*.

[14] Kovalenko A., Chable-Bessia C., Cantarella G., Israël A., Wallach D., Courtois G. (2003). The tumour suppressor CYLD negatively regulates NF-*κ*B signalling by deubiquitination. *Nature*.

[15] Trompouki E., Hatzivassillou E., Tsichritzis T., Farmer H., Ashworth A., Mosialos G. (2003). CYLD is a deubiquitinating enzyme that negatively regulates NF-*κ*B activation by TNFR family members. *Nature*.

[16] Nikolaou K., Tsagaratou A., Eftychi C., Kollias G., Mosialos G., Talianidis I. (2012). Inactivation of the Deubiquitinase CYLD in Hepatocytes Causes Apoptosis, Inflammation, Fibrosis, and Cancer. *Cancer Cell*.

[17] Sun S.-C. (2008). Deubiquitylation and regulation of the immune response. *Nature Reviews Immunology*.

[18] Lim J.-H., Ha U.-H., Woo C.-H., Xu H., Li J.-D. (2008). CYLD is a crucial negative regulator of innate immune response in Escherichia coli pneumonia. *Cellular Microbiology*.

[19] Hellerbrand C., Bumes E., Bataille F., Dietmaier W., Massoumi R., Bosserhoff A. K. (2007). Reduced expression of CYLD in human colon and hepatocellular carcinomas. *Carcinogenesis*.

[20] Zhang J., Stirling B., Temmerman S. T. (2006). Impaired regulation of NF-*κ*B and increased susceptibility to colitis-associated tumorigenesis in CYLD-deficient mice. *The Journal of Clinical Investigation*.

[21] Massoumi R., Chmielarska K., Hennecke K., Pfeifer A., Fässler R. (2006). Cyld inhibits tumor cell proliferation by blocking Bcl-3-dependent NF- kappaB signaling. *Cell*.

[22] O'Donnell M. A., Perez-Jimenez E., Oberst A. (2011). Caspase 8 inhibits programmed necrosis by processing CYLD. *Nature Cell Biology*.

[23] Hövelmeyer N., Wunderlich F. T., Massoumi R. (2007). Regulation of B cell homeostasis and activation by the tumor suppressor gene CYLD. *The Journal of Experimental Medicine*.

[24] Urbanik T., Boger R. J., Longerich T. (2012). Liver specific deletion of CYLDexon7/8 induces severe biliary damage, fibrosis and increases hepatocarcinogenesis in mice. *Journal of Hepatology*.

[25] Brummelkamp T. R., Nijman S. M. B., Dirac A. M. G., Bernards R. (2003). Loss of the cylindromatosis tumour suppressor inhibits apoptosis by activating NF-*κ*B. *Nature*.

[26] Urbanik T., Koehler B. C., Wolpert L. (2014). CYLD deletion triggers nuclear factor-KB-signaling and increases cell death resistance in murine hepatocytes. *World Journal of Gastroenterology*.

[27] Lim S. M., Jeong Y., Lee S. (2015). Identification of *β*-lapachone analogs as novel MALT1 inhibitors to treat an aggressive subtype of diffuse large B-cell lymphoma. *Journal of Medicinal Chemistry*.

[28] Bonapace L., Bornhauser B. C., Schmitz M. (2010). Induction of autophagy-dependent necroptosis is required for childhood acute lymphoblastic leukemia cells to overcome glucocorticoid resistance. *The Journal of Clinical Investigation*.

